# Mediterranean diet adherence in pediatric familial Mediterranean fever: clinical and inflammatory correlates

**DOI:** 10.3389/fped.2026.1917680

**Published:** 2026-08-11

**Authors:** Hande Ilgaz Tüzen, Tuncay Aydın, Zehra Kızıldağ, Rana İşgüder, Rüya Torun, Erbil Ünsal, Balahan Bora

**Affiliations:** 1 Department of Pediatric Rheumatology, Izmir City Hospital, Izmir, Türkiye; 2 Department of Pediatric Rheumatology, Atatürk University Faculty of Medicine, Erzurum, Türkiye; 3 Department of Pediatric Rheumatology, Trakya University Faculty of Medicine, Edirne, Türkiye; 4 Department of Pediatric Rheumatology, Adana City Hospital, Adana, Türkiye; 5 Department of Pediatric Rheumatology, Dokuz Eylül University Faculty of Medicine, Izmir, Türkiye

**Keywords:** autoinflammatory diseases, familial Mediterranean fever, feeding behavior, inflammation, mediterranean diet

## Abstract

Familial Mediterranean fever is an autoinflammatory disease characterized by recurrent episodes of fever and serositis, with subclinical inflammation that may persist between attacks. Although the Mediterranean diet has recognized anti-inflammatory properties, its clinical relevance in pediatric familial Mediterranean fever remains unclear. In this cross-sectional observational study, children aged 7–18 years with a confirmed diagnosis of familial Mediterranean fever who were followed at the Pediatric Rheumatology Clinic of Dokuz Eylül University between June 2022 and February 2023 were evaluated using standardized dietary questionnaires during outpatient visits. A total of 91 patients met the inclusion criteria. Associations between Mediterranean diet adherence, eating behaviors, attack frequency, disease severity, and selected clinical and inflammatory parameters were analyzed using appropriate comparative statistical tests. Eating behavior showed nominal associations with body mass index and exertional leg pain. Leukocyte count differed across the KIDMED categories in the overall comparison; however, no pairwise comparison remained statistically significant after Bonferroni correction. Neither dietary measure was associated with attack frequency or disease severity. In pediatric familial Mediterranean fever, Mediterranean diet adherence and eating behaviors showed nominal exploratory associations with selected clinical and inflammatory parameters but were not associated with attack frequency or overall disease severity. These findings suggest that dietary patterns may be more closely related to inter-attack clinical and inflammatory expression than to acute disease activity and may help inform future prospective studies on modifiable lifestyle factors in pediatric autoinflammatory disease.

## Introduction

Familial Mediterranean fever (FMF) is the most common hereditary autoinflammatory disease, characterized by recurrent, self-limiting episodes of fever and serositis, including peritonitis, pleuritis, and pericarditis (1–3). Although attacks are generally transient, persistent subclinical inflammation may occur between episodes and contribute to cumulative disease burden and long-term complications, particularly AA amyloidosis (4, 5). FMF is associated with pathogenic variants in the Mediterranean fever (*MEFV*) gene, which encodes pyrin. The clinical expression of the disease demonstrates substantial genotype-phenotype heterogeneity. Commonly reported *MEFV* variants include M694V, M680I, V726A, M694I, and E148Q, although the penetrance and pathogenic significance of E148Q remain less clearly defined (6, 7). Exon 10 variants, particularly biallelic M694V, have been associated with more severe disease and an increased risk of amyloidosis. M694V homozygosity has also been linked to colchicine-resistant disease in children (7, 8). These findings indicate that genetic background is an important determinant of disease severity and treatment response in FMF.

At the molecular level, pathogenic *MEFV* variants lead to dysregulated pyrin-inflammasome activation and increased production of proinflammatory cytokines, particularly interleukin-1 beta (IL-1β) (3, 9, 10). Acute-phase reactants, including C-reactive protein (CRP), serum amyloid A (SAA), and erythrocyte sedimentation rate (ESR), increase markedly during attacks, while low-grade inflammation may persist during clinically asymptomatic periods (3–5). Although the primary inflammatory mechanisms of FMF are genetically determined, variability in disease expression cannot be explained by genotype alone. Environmental and lifestyle-related factors may also influence inflammatory burden, attack perception, treatment response, and inter-attack clinical manifestations. Identifying potentially modifiable factors is therefore relevant to the broader management of FMF. Dietary factors represent one such potentially modifiable domain. Previous studies have evaluated patient-reported food triggers, salt- and fat-related food preferences, antioxidant-rich diets, and anti-inflammatory dietary interventions in patients with FMF (11–14). However, the reported associations with attack frequency, colchicine response, inflammatory markers, disease severity, and patient-reported outcomes have been inconsistent.

The Mediterranean diet is characterized by a high intake of vegetables, fruits, legumes, whole grains, nuts, olive oil, and fish, together with limited consumption of processed foods and saturated fats. Its potential anti-inflammatory effects have been attributed to dietary fiber, monounsaturated and omega-3 fatty acids, antioxidant compounds, and polyphenols (15, 16). Greater adherence to this dietary pattern has been associated with lower levels of several proinflammatory biomarkers and with regulation of inflammation-related epigenetic pathways (15, 16). These biological effects may be relevant to FMF, in which subclinical inflammation can persist between attacks, and support the investigation of Mediterranean dietary patterns as potentially modifiable adjuncts to standard pharmacological treatment (4, 5). Despite this biological rationale, evidence regarding Mediterranean diet adherence in FMF remains limited. A recent study evaluating Mediterranean diet adherence in an FMF cohort found no association with overall disease severity, although differences were observed in selected clinical manifestations (17). Moreover, previous studies have predominantly involved adult populations or have examined isolated dietary preferences rather than adherence to an overall Mediterranean dietary pattern (11–14).

Whether Mediterranean diet adherence and food-choice behavior are associated with attack burden, disease severity, clinical manifestations, or inter-attack inflammatory parameters in children with FMF therefore remains unclear. Given the genotype–phenotype heterogeneity of FMF, genetic background should also be considered when interpreting possible relationships between dietary factors and disease expression. The primary aim of the study was to assess the association between Mediterranean diet adherence and the number of FMF attacks during the previous year in a pediatric FMF cohort. The secondary aims were to examine the relationships of Mediterranean diet adherence and food-choice behavior with disease severity, clinical manifestations, and selected inter-attack inflammatory parameters. These findings are expected to contribute to a better understanding of the clinical relevance of modifiable lifestyle factors in pediatric FMF and to provide a basis for future prospective studies.

## Materials and methods

### Ethics approval and consent to participate

This study was approved by the Dokuz Eylül University Non-Interventional Research Ethics Committee (approval date: 18 May 2022; protocol number: 2022/18–02). Written informed consent was obtained from the parents or legal guardians of all participating children, and assent was obtained from the children when appropriate. All procedures were conducted in accordance with the Declaration of Helsinki and relevant institutional ethical guidelines.

### Study design and setting

This cross-sectional observational study was conducted at the Pediatric Rheumatology Clinic of Dokuz Eylül University Hospital between June 2022 and February 2023. The study population consisted of pediatric patients aged 7–18 years who had been diagnosed with FMF according to the Tel-Hashomer criteria (18).

Patients were eligible for inclusion if they were not experiencing an active FMF attack at the time of assessment and had remained attack-free for at least 2 weeks. The requirement for a minimum 2-week attack-free period was defined as a pragmatic criterion to reduce the short-term inflammatory effects of acute attacks and to ensure that evaluations were performed during a clinically stable period.

Patients receiving non-FMF medications with the potential to alter systemic inflammatory status were excluded. FMF-directed treatment was permitted and was not considered an exclusion criterion. All patients were receiving colchicine at the time of assessment, and patients with colchicine-resistant disease could additionally receive anti-interleukin-1 biologic therapy. No patient received biologic therapy without concomitant colchicine. The present analysis focused on the validated composite KIDMED and FBS scores and did not examine individual questionnaire items. In addition, patients with concomitant chronic inflammatory or autoimmune diseases, chronic infections, endocrine or metabolic disorders, chronic renal or hepatic diseases, malignancy, or neurological disorders requiring long-term medical treatment were excluded because these conditions could independently affect inflammatory status, dietary habits, or growth parameters. The study was reported in accordance with the Strengthening the Reporting of Observational Studies in Epidemiology (STROBE) guidelines (19).

### Variables

The primary exposure variables were Mediterranean diet adherence, assessed using the Mediterranean Diet Quality Index for Children and Adolescents (KIDMED) (20, 21) and the food-choice behavior, assessed using the Food Behavior Scale (FBS) (22, 23). The primary outcome variable was the number of FMF attacks during the previous year. Secondary outcomes included disease severity score, body mass index (BMI), selected clinical manifestations (fever, abdominal pain, arthritis, chest pain, and exertional leg pain), inflammatory markers (CRP and leukocyte count) and colchicine resistance.

Additional demographic and clinical variables included age at assessment, sex, family history of FMF, age at symptom onset, age at diagnosis, diagnostic delay, follow-up duration, current colchicine dose, and concomitant anti-interleukin-1 therapy. *MEFV* genotypes were classified into four mutually exclusive categories according to the number and reported zygosity of the identified variants: single-variant genotype, homozygous genotype, two-variant genotype, and complex/multivariant genotype. The single-variant category included patients with one documented *MEFV* variant and no second reported variant. The homozygous category included patients in whom the same variant was reported on both alleles. The two-variant category included patients with two different documented variants. The complex/multivariant category included genotypes containing three or more reported variant alleles or multiple zygosity combinations, including double homozygosity. M694V homozygosity was additionally evaluated as a separate binary variable because of its clinical relevance in FMF. Associations of M694V homozygosity with disease severity and colchicine resistance were evaluated as exploratory analyses. Abdominal pain was recorded as an FMF-related clinical manifestation. Other gastrointestinal symptoms, including diarrhea, constipation, nausea, vomiting, and abdominal bloating, were not systematically collected and therefore could not be included in the analyses.

### Data sources and measurements

Demographic, clinical, genetic, treatment-related, and laboratory data were obtained from the electronic medical record system of Dokuz Eylül University Hospital, archived outpatient records, and structured study forms. Laboratory parameters were obtained from the most recent attack-free outpatient visit.

Mediterranean diet adherence was assessed using the validated Turkish version of the 16-item KIDMED (20, 21). Items consistent with the Mediterranean dietary pattern are scored +1, whereas unfavorable dietary items are scored −1. Scores were classified as very low adherence (≤3; Group A), intermediate adherence requiring improvement (4–7; Group B), or optimal adherence (≥8; Group C). Food-choice behavior was assessed using the validated Turkish version of the 14-item pictorial FBS (22, 23). Each item requires a forced choice between two foods differing primarily in fat and sodium content; the healthier choice is scored +1 and the less healthy choice −1. Higher scores indicate healthier food-choice behavior. For the present analysis, scores ≤0 were classified as Group I, representing less favorable eating behavior, and scores >0 as Group II, representing more favorable eating behavior. During the study visit, the Turkish versions of the KIDMED and FBS were completed by the child, with assistance from an accompanying parent or legal guardian when required for comprehension. Both questionnaires were completed during the same study visit and checked for completeness before scoring.

BMI and BMI percentiles were calculated according to the Centers for Disease Control and Prevention growth charts (24). Disease severity was assessed using the Pras scoring system (25). Colchicine resistance was defined as at least one FMF attack per month despite confirmed treatment adherence and use of the maximally tolerated colchicine dose for at least 6 months, in accordance with the EULAR recommendations (26).

### Statistical analysis

Statistical analyses were performed using IBM SPSS Statistics (version 24.0). The distribution of continuous variables was assessed within the relevant KIDMED and FBS subgroups using the Shapiro–Wilk test together with visual inspection of histograms and quantile-quantile plots. Continuous variables with a normal distribution are presented as mean ± standard deviation, whereas non-normally distributed variables are presented as median [interquartile range]. Categorical variables are expressed as counts and percentages (%). For comparisons between two groups, normally distributed continuous variables were analyzed using the independent-samples *t*-test, whereas non-normally distributed continuous variables were analyzed using the Mann–Whitney *U*-test. Categorical variables were compared using the Pearson chi-square test; Fisher's exact test was used when expected cell counts were low.

For comparisons among three groups, normally distributed continuous variables were analyzed using one-way analysis of variance (ANOVA), whereas non-normally distributed continuous variables were analyzed using the Kruskal–Wallis test. Categorical variables were compared using the Pearson chi-square test, and the Fisher exact test was applied when appropriate. When an overall significant difference was identified in three-group comparisons, pairwise *post hoc* comparisons were performed with Bonferroni correction. Because the analyses of secondary clinical and laboratory outcomes were exploratory, no multiplicity correction was applied across the complete family of secondary comparisons. Bonferroni correction was restricted to *post hoc* pairwise comparisons following a statistically significant three-group omnibus test. For the four-level *MEFV* genotype-pattern comparison, pairwise *post hoc* analyses following a significant omnibus KIDMED comparison were performed using Monte Carlo Fisher-Freeman-Halton exact tests with Holm correction. In exploratory variant-specific analyses, the presence of any M694V-containing genotype and M694V homozygosity were compared across the KIDMED categories using Pearson's chi-square test, and a Cochran-Armitage test for trend was additionally performed for M694V carriage. In all other analyses, a two-sided *p* value of <0.05 was considered statistically significant.

Effect sizes with 95% confidence intervals (CI) were calculated to complement the hypothesis-test results. Odds ratios (OR) were used for categorical comparisons between the two FBS groups, whereas Cramér's V was used for categorical comparisons across the three KIDMED groups. For continuous variables, Hedges' g and rank-biserial correlation coefficients were calculated for parametric and nonparametric two-group comparisons, respectively. Omega squared and rank epsilon squared were calculated for parametric and nonparametric three-group comparisons, respectively. Confidence intervals for rank epsilon squared were estimated using 5,000 bootstrap iterations. Effect-size analyses were performed using RStudio (version 2025.09.2+418). Because the proportion of missing observations in the dataset was generally low (approximately 5%), no imputation method was applied; instead, missing data were handled by excluding incomplete observations from the relevant analyses only. Analyses based on KIDMED and FBS scores were conducted separately to evaluate different dimensions of dietary adherence and eating behavior.

To assess the statistical capacity of the available sample size, a *post hoc* sensitivity power analysis was performed using RStudio (version 2025.09.2+418). Assuming a two-sided *α* of 0.05 and a target power of 80%, the available sample size was estimated to detect a minimum effect size of approximately Cohen's d = 0.59 or greater for pairwise comparisons between the FBS groups.

## Results

A total of 95 patients were screened; 4 were excluded because of missing genetic or clinical data at the time of diagnosis, and 91 patients were included in the study. Patients were categorized into three groups according to their KIDMED scores: Group A (≤3), Group B (4–7) and Group C (≥8). All patients were receiving colchicine at the time of assessment. Seventy-three patients (80.2%) were treated with colchicine alone, whereas 18 (19.8%) received concomitant anti–interleukin-1 biologic therapy for colchicine-resistant disease. No patient received biologic therapy without concomitant colchicine. Demographic and clinical characteristics according to the KIDMED categories are presented in Table 1. No statistically significant differences were observed among the KIDMED groups in age at assessment, sex, family history of FMF, M694V homozygosity, age at symptom onset, age at diagnosis, diagnostic delay, follow-up duration, BMI, BMI percentile, disease severity, attack frequency, colchicine dose, selected clinical manifestations, or colchicine resistance (Table 1).

**Table 1:** Demographic and clinical characteristics according to KIDMED categories.

Variables	Overall cohort (*n* = 91)	Group A (*n* = 33)	Group B (*n* = 30)	Group C (*n* = 28)	Effect Size (95% CI)	*p*-value
Age at assessment, years	11 [10–14]	11 [9–14]	12 [11–14]	10 [10–13]	0.04 (0.00–0.16)	0.18
Female sex, *n* (%)	35 (38.5)	13 (39.3)	12 (40.0)	10 (35.7)	0.04 (0.00–0.15)	0.78
Family history of FMF, *n* (%)	56 (61.5)	21 (63.0)	19 (63.3)	16 (57.1)	0.06 (0.00–0.21)	0.62
M694V homozygous genotype, *n* (%)	25 (27.5)	6 (18.2)	10 (33.3)	9 (32.1)	0.16 (0.00–0.34)	0.32
Age at symptom onset (months)	59 [27–89]	60 [27–98]	71 [39–89]	46 [29–69]	0.04 (0.00–0.15)	0.20
Age at diagnosis (months)	75 [48–112]	82 [59–128]	91 [49–117]	62 [48–87]	0.03 (0.00–0.15)	0.25
Delay in diagnosis (months)	11 [6–25]	13 [6–25]	11 [7–22]	13 [6–32]	0.01 (0.00–0.10)	0.72
Follow-up duration (months)	50 [16–77]	39 [10–68]	38 [15–69]	64 [36–92]	0.06 (0.01–0.19)	0.08
BMI (kg/m^2^)	19.5 ± 4.3	19.3 ± 4.2	19.9 ± 4.5	19.0 ± 4.2	0.00 (0.00–0.00)	0.74
BMI percentile (≥85%)	26 (28.6)	9 (27.2)	9 (30.0)	8 (28.5)	0.03 (0.00–0.05)	0.91
Pras disease severity score	6.6 ± 2.2	6.7 ± 2.2	6.5 ± 1.8	6.6 ± 2.3	0.00 (0.00–0.00)	0.96
Number of attacks in the previous year	1 [0–3]	1 [0–3]	0 [0–3]	1 [0–2]	0.01 (0.00–0.11)	0.70
Colchicine dose (mg/kg/day)	0.024 ± 0.013	0.02 ± 0.01	0.02 ± 0.01	0.02 ± 0.00	0.00 (0.00–0.00)	0.44
Fever, *n* (%)	61 (67.0)	22 (66.6)	21 (70.0)	18 (64.2)	0.05 (0.00–0.18)	0.86
Abdominal pain, *n* (%)	79 (86.8)	29 (87.8)	26 (86.6)	24 (85.7)	0.03 (0.00–0.07)	0.81
Chest pain, *n* (%)	9 (9.9)	1 (3.0)	6 (20.0)	2 (7.1)	0.24 (0.00–0.43)	0.52
Arthritis, *n* (%)	21 (23.1)	7 (21.2)	9 (30.0)	5 (17.8)	0.12 (0.00–0.29)	0.80
Exertional leg pain, *n* (%)	46 (50.5)	18 (54.5)	15 (50.0)	13 (46.4)	0.07 (0.00–0.22)	0.53
Colchicine resistance, *n* (%)	18 (19.8)	5 (15.1)	7 (23.3)	6 (21.4)	0.09 (0.00–0.26)	0.52

KIDMED categories were defined as follows: Group A, very low adherence (score≤3); Group B, intermediate adherence requiring improvement (score 4–7); and Group C, optimal adherence (score≥8).

BMI, Body Mass Index; CI, Confidence interval; FMF, Familial Mediterranean Fever; KIDMED, Mediterranean Diet Quality Index.

Data are presented as mean ± standard deviation, median [interquartile range], or *n* (%), as appropriate. Continuous variables were compared using one-way ANOVA or the Kruskal–Wallis test, and categorical variables were compared using the Pearson chi-square or Fisher exact test. Omega squared, rank epsilon squared, and Cramér's V were reported as effect-size measures for parametric continuous, nonparametric continuous, and categorical variables, respectively.

Patients were divided into two groups according to their FBS scores: Group I (≤0) represents less favorable eating behavior, whereas Group II (>0) represents more favorable eating behavior. Demographic and clinical characteristics according to the FBS groups are presented in Table 2. No statistically significant differences were observed in the remaining demographic or clinical variables. Exertional leg pain was more frequent in Group I than in Group II (61.7% vs. 38.6%; *p* = 0.04; OR = 2.56, 95% CI 1.10–5.96). BMI was also higher in Group I than in Group II (20.5 ± 3.8 vs. 18.3 ± 4.5 kg/m²; *p* = 0.02; Hedges' g = 0.51, 95% CI 0.10–0.93) (Table 2).

**Table 2:** Demographic and clinical characteristics according to FBS groups.

Variables	Overall cohort (*n* = 91)	Group I (*n* = 47)	Group II (*n* = 44)	Effect Size (95% CI)	*p*-value
Age at assessment, years	11 [10–14]	12 [10–14]	11 [10–14]	−0.03 (−0.27–0.21)	0.80
Female sex, *n* (%)	35 (38.5)	17 (36.1)	18 (40.9)	0.82 (0.35–1.91)	0.67
Family history of FMF, *n* (%)	56 (61.5)	31 (65.9)	25 (56.8)	1.47 (0.63–3.44)	0.40
M694V homozygous genotype, *n* (%)	25 (27.5)	13 (27.6)	12 (27.2)	1.02 (0.41–2.56)	0.97
Age at symptom onset (months)	59 [27–89]	60 [33–88]	54 [24–88]	0.06 (−0.17–0.29)	0.61
Age at diagnosis (months)	75 [48–112]	76 [47–100]	75 [51–120]	−0.09 (−0.32–0.15)	0.47
Delay in diagnosis (months)	11 [6–25]	10 [6–24]	12 [7–31]	−0.11 (−0.33–0.13)	0.38
Follow-up duration (months)	50 [16–77]	57 [25–77]	38 [16–73]	0.09 (−0.15–0.31)	0.49
BMI (kg/m²)	19.5 ± 4.3	20.5 ± 3.8	18.3 ± 4.5	0.51 (0.10–0.93)	0.02
BMI percentile (≥85%)	26 (28.6)	16 (34.0)	10 (22.7)	1.75 (0.69–4.44)	0.24
Pras disease severity score	6.6 ± 2.2	6.8 ± 2.2	6.5 ± 2.0	0.17 (−0.24–0.58)	0.41
Number of attacks in the previous year	1.0 [0.0–3.0]	1.0 [0.0–3.0]	0.0 [0.0–2.2]	0.04 (−0.20–0.27)	0.82
Colchicine dose (mg/kg/day)	0.024 ± 0.013	0.023 ± 0.014	0.024 ± 0.011	−0.09 (−0.52–0.33)	0.66
Fever, *n* (%)	61 (67.0)	32 (68.0)	29 (65.9)	1.10 (0.46–2.65)	0.83
Abdominal pain, *n* (%)	79 (86.8)	42 (89.3)	37 (84.0)	1.59 (0.46–5.44)	0.54
Chest pain, *n* (%)	9 (9.9)	5 (10.6)	4 (9.09)	1.19 (0.30–4.75)	0.81
Arthritis, *n* (%)	21 (23.1)	14 (29.7)	7 (15.9)	2.24 (0.81–6.23)	0.14
Exertional leg pain, *n* (%)	46 (50.5)	29 (61.7)	17 (38.6)	2.56 (1.10–5.96)	0.04
Colchicine resistance, *n* (%)	18 (19.8)	12 (25.5)	6 (13.6)	2.17 (0.74–6.41)	0.19

Higher FBS scores indicate healthier food choices. Group I included patients with scores ≤0, representing less favorable eating behavior, and Group II included patients with scores >0, representing more favorable eating behavior.

BMI, Body Mass Index; CI, Confidence interval; FBS, Food Behavior Scale; FMF, Familial Mediterranean Fever; OR, Odds ratio.

Data are presented as mean ± standard deviation, median [interquartile range], or *n* (%), as appropriate. Continuous variables were compared using the independent-samples t-test or Mann–Whitney *U*-test, and categorical variables were compared using the Pearson chi-square or Fisher exact test. Hedges' g, rank-biserial correlation, and ORs were reported as effect-size measures for parametric continuous, nonparametric continuous, and binary categorical variables, respectively.

Laboratory characteristics according to the KIDMED categories are presented in Table 3. Leukocyte count differed among the three groups in the overall comparison (*p* = 0.04; *ω*^2^ = 0.05, 95% CI 0.00–0.15); however, no pairwise comparison remained statistically significant after Bonferroni correction. No significant differences were observed in the other laboratory parameters. The distribution of the four-level *MEFV* genotype pattern differed among the KIDMED categories (*p* = 0.043; Cramér's V = 0.27, 95% CI 0.00–0.37). Single-variant genotypes were more frequent in Group A, whereas two-variant and complex/multivariant genotypes were descriptively more frequent in Groups B and C. However, none of the pairwise comparisons remained statistically significant after Holm correction; the smallest adjusted *p* value was 0.064 for the comparison between Groups A and C (Table 3). The specific *MEFV* genotype combinations included within the four-level genotype classification and their frequencies in the overall cohort are presented in Supplementary Table S1. The most frequent specific genotype combinations were M694V/M694V (12/91, 13.2%), M694 V + R202Q (8/91, 8.8%), R202Q as a single documented variant (8/91, 8.8%), and M694 V/M694V + R202Q/R202Q (7/91, 7.7%). In exploratory variant-specific analyses, the prevalence of M694V-containing genotypes differed across the KIDMED categories. An M694V variant was present in 15 of 33 patients (45.5%) in Group A, 18 of 30 (60.0%) in Group B, and 22 of 28 (78.6%) in Group C (*p* = 0.031; Cramér's V = 0.28, 95% CI 0.00–0.47). The Cochran-Armitage trend test demonstrated an increasing prevalence of M694V-containing genotypes across progressively higher KIDMED categories (*p* = 0.01). In contrast, M694V homozygosity was present in 6 of 33 patients (18.2%) in Group A, 10 of 30 (33.3%) in Group B, and 9 of 28 (32.1%) in Group C and did not differ significantly among the KIDMED categories (*p* = 0.32; Cramér's V = 0.16, 95% CI 0.00–0.34).

**Table 3:** Laboratory characteristics and genotype pattern according to KIDMED categories.

Variables	Overall cohort (*n* = 91)	Group A (*n* = 33)	Group B (*n* = 30)	Group C (*n* = 28)	Effect size (95% CI)	*p*-value
Hemoglobin (g/dL)	13.1 ± 1.4	13.0 ± 1.4	13.2 ± 1.3	13.0 ± 1.4	0.00 (0.00–0.00)	0.70
Leukocyte count (×10³/µL)	6.7 ± 2.0	7.2 ± 2.5	6.8 ± 1.7	5.9 ± 1.4	0.05 (0.00–0.15)	0.04
Neutrophil/Lymphocyte ratio (NLR)	1.4 [1.0–1.8]	1.4 [1.0–2.3]	1.4 [1.0–1.8]	1.4 [1.1–1.6]	0.01 (0.00–0.10)	0.74
Platelets (×10³/µL)	293 ± 79	305 ± 74	285 ± 76	285 ± 85	0.00 (0.00–0.00)	0.52
Mean platelet volume (fL)	8.6 ± 1.0	8.5 ± 1.1	8.6 ± 0.9	8.7 ± 0.7	0.00 (0.00–0.00)	0.69
CRP (mg/L)	1.0 [0.5–4.5]	1.7 [0.4–6.0]	1.0 [0.5–2.3]	1.1 [0.5–4.7]	0.01 (0.00–0.11)	0.78
*MEFV* genotype pattern						
Single-variant genotype	29 (31.9)	15 (45.5)	9 (30.0)	5 (17.9)	0.27 (0.00–0.37)	0.043
Homozygous genotype	17 (18.7)	7 (21.2)	7 (23.3)	3 (10.7)		
Two-variant genotype	28 (30.8)	9 (27.3)	6 (20.0)	13 (46.4)		
Complex/multivariant genotype	17 (18.7)	2 (6.1)	8 (26.7)	7 (25.0)		

KIDMED categories were defined as follows: Group A, very low adherence (score ≤3); Group B, intermediate adherence requiring improvement (score 4–7); and Group C, optimal adherence (score ≥8).

CI, Confidence interval; CRP, C-Reactive Protein; KIDMED, Mediterranean Diet Quality Index; *MEFV*, Mediterranean fever gene; NLR, neutrophil-to-lymphocyte ratio.

Data are presented as mean ± standard deviation, median [interquartile range], or *n* (%), as appropriate. Overall comparisons were performed using one-way ANOVA or Kruskal–Wallis test according to the distribution of the data. No multiplicity correction was applied across the complete family of secondary comparisons. Bonferroni correction was used only for *post hoc* pairwise comparisons following a significant three-group omnibus test. Although leukocyte levels differed significantly in the overall comparison (*p* = 0.04), no pairwise comparison remained statistically significant after Bonferroni correction. Omega squared was calculated for variables compared using one-way analysis of variance, and rank epsilon squared was calculated for variables compared using the Kruskal–Wallis test. Omega-squared estimates below zero after bias correction were bounded at zero. CIs for rank epsilon squared were estimated using 5,000 bootstrap iterations. Genotype patterns were classified conservatively as single-variant, homozygous, two-variant, or complex/multivariant genotypes. The overall genotype-pattern distribution was compared using Pearson's chi-square test, and Cramér's V was reported as the effect-size measure. Pairwise comparisons were performed using Monte Carlo Fisher-Freeman-Halton tests with Holm adjustment. Group A vs. Group C: Monte Carlo exact *p* ≈ 0.021; Holm-adjusted *p* ≈ 0.064.

Laboratory characteristics according to the FBS groups are presented in Table 4. No statistically significant differences were observed between the groups in hemoglobin, leukocyte count, neutrophil-to-lymphocyte ratio, platelet count, mean platelet volume, or CRP (Table 4). The overall *MEFV* genotype-pattern distribution did not differ significantly between the FBS groups (Table 4).

**Table 4:** Laboratory characteristics and genotype pattern according to FBS groups.

Variables	**Overall cohort (*n*** **=** **91)**	**Group I (*n*** **=** **47)**	**Group II (*n*** **=** **44)**	**Effect size (95% CI)**	***p*-value**
Hemoglobin (g/dL)	13.1 ± 1.4	13.1 ± 1.4	13.0 ± 1.3	0.12 (−0.28–0.53)	0.55
Leukocyte count (×10³/µL)	6.7 ± 2.0	6.6 ± 2.0	6.7 ± 2.0	−0.06 (−0.47–0.35)	0.77
Neutrophil/Lymphocyte ratio (NLR)	1.4 [1.0–1.8]	1.4 [1.0–2.3]	1.3 [1.0–1.6]	0.06 (−0.18–0.29)	0.64
Platelets (×10^3^/µL)	293 ± 79	285 ± 70	300 ± 86	−0.19 (−0.60–0.22)	0.36
Mean platelet volume (fL)	8.6 ± 1.0	8.7 ± 0.9	8.5 ± 1.0	0.24 (−0.17–0.65)	0.25
CRP (mg/L)	1.0 [0.5–4.5]	1.3 [0.6–4.0]	0.8 [0.4–4.4]	0.15 (−0.09–0.37)	0.21
*MEFV* genotype pattern					
Single-variant genotype	29 (31.9)	12 (25.5)	17 (38.6)	0.18 (0.00–0.34)	0.413
Homozygous genotype	17 (18.7)	11 (23.4)	6 (13.6)		
Two-variant genotype	28 (30.8)	16 (34.0)	12 (27.3)		
Complex/multivariant genotype	17 (18.7)	8 (17.0)	9 (20.5)		

Higher FBS scores indicate healthier food choices. Group I included patients with scores ≤0, representing less favorable eating behavior, and Group II included patients with scores >0, representing more favorable eating behavior.

CI, Confidence interval; CRP, C-Reactive Protein; FBS, Food Behavior Scale; *MEFV*, Mediterranean fever gene; NLR, neutrophil-to-lymphocyte ratio.

Data are presented as mean ± standard deviation, median [interquartile range], or *n* (%), as appropriate. *p* values were calculated using the independent-samples t-test or Mann–Whitney *U*-test according to distribution of the data. Hedges' g was calculated for variables compared using the independent-samples *t*-test, and rank-biserial correlation was calculated for variables compared using the Mann–Whitney *U*-test. Positive values indicate higher means or ranks in FBS Group I than in Group II. Genotype patterns were classified as single-variant, homozygous, two-variant, or complex/multivariant genotypes. The overall distribution was compared using Pearson's chi-square test, and Cramér's V was reported as the effect-size measure.

In the exploratory genotype analyses, M694 V homozygosity was present in 12 of 18 patients (66.7%) with severe disease, defined as a Pras score ≥9, compared with 13 of 73 patients (17.8%) with mild-to-moderate disease, defined as a Pras score <9 (OR = 9.23, 95% CI 2.93–29.12; *p* < 0.001). It was also present in 14 of 18 patients (77.8%) with colchicine resistance and in 11 of 73 patients (15.1%) without colchicine resistance (OR = 19.73, 95% CI 5.47–71.16; *p* < 0.001).

## Discussion

In this cross-sectional study, Mediterranean diet adherence and food-choice behavior were not associated with attack frequency or disease severity in pediatric patients with FMF. However, exploratory differences were observed in BMI and exertional leg pain according to FBS groups and in leukocyte count according to KIDMED categories. The distribution of the four-level *MEFV* genotype pattern and the prevalence of M694V-containing genotypes also differed across the KIDMED categories. These findings suggest that dietary patterns may be more closely related to selected inter-attack clinical and inflammatory characteristics than to acute attack burden or overall disease severity. Nevertheless, because of the cross-sectional design and the absence of multiplicity adjustment across the complete family of secondary analyses, these associations should be considered hypothesis-generating and should not be interpreted as causal.

The absence of a significant association between Mediterranean diet adherence and attack frequency or disease severity is consistent with the biological nature of FMF, in which acute inflammatory attacks are primarily driven by dysregulated pyrin inflammasome activation (3, 9, 10). Accordingly, dietary patterns may be more closely related to the inflammatory burden during attack-free periods than to the dynamics of acute attacks. Previous studies evaluating dietary factors in FMF have reported heterogeneous findings (11–14, 17, 27–31). In a pediatric cross-sectional study, Ekinci et al. reported a higher frequency of complete colchicine response among patients with healthier low-salt and low-fat food preferences, whereas attack frequency, disease severity, and acute-phase reactants were similar between the dietary groups (12). More recently, Hammoud et al. found no association between Mediterranean diet adherence and overall FMF severity, although selected clinical manifestations differed across adherence categories (17). A study of adolescents with FMF also identified differences in dietary inflammatory index, oxidative stress, and selected inflammatory parameters, further suggesting that relationships between nutrition and FMF may vary according to the dietary measure and outcome evaluated (31). Taken together, these findings support the possibility that dietary behavior may be more closely related to selected clinical or inflammatory characteristics than to the principal determinants of attack frequency and disease severity.

The genotype analyses provide additional context for interpreting the comparisons across KIDMED categories. The distribution of the four-level *MEFV* genotype pattern differed in the overall comparison, with single-variant genotypes occurring more frequently in the very-low-adherence group and two-variant or complex/multivariant genotypes occurring descriptively more frequently in the intermediate- and optimal-adherence groups. However, none of the pairwise comparisons remained statistically significant after Holm correction. In the exploratory variant-specific analysis, the prevalence of M694V-containing genotypes increased across progressively higher KIDMED categories, whereas M694V homozygosity did not differ significantly among the groups. Because *MEFV* genotype is inherited and cannot be influenced by current dietary behavior, this finding should not be interpreted as an effect of Mediterranean diet adherence on genotype distribution. It may instead reflect chance imbalance, the composition of this single-center cohort, familial or sociodemographic characteristics associated with dietary behavior, or other unmeasured confounding factors. The absence of corresponding differences in attack frequency and disease severity across the KIDMED groups further argues against a direct clinical interpretation of this exploratory association. M694V homozygosity was strongly associated with severe disease and colchicine resistance. This finding is consistent with pediatric genotype–phenotype studies identifying M694V homozygosity as a marker of a more severe phenotype and an increased risk of colchicine-resistant disease (8). Therefore, the observed genotype imbalance reinforces the importance of accounting for clinically relevant *MEFV* variants when evaluating associations between dietary factors and FMF expression. Larger studies should consider genotype-adjusted or genotype-stratified analyses to distinguish dietary associations from the effects of underlying genetic background.

Leukocyte count differed across the KIDMED categories in the overall comparison and was highest among patients with very low Mediterranean diet adherence. However, no pairwise comparison remained statistically significant after Bonferroni correction. Furthermore, because no multiplicity correction was applied across the complete family of secondary analyses, the possibility of a chance finding cannot be excluded. This result should therefore be considered a hypothesis-generating observation rather than confirmatory evidence of a relationship between Mediterranean diet adherence and low-grade inflammatory burden. By contrast, CRP did not differ according to either dietary measure. Subclinical inflammation may persist during clinically asymptomatic periods in FMF, but individual acute-phase reactants do not identify it with equal sensitivity (4, 5). In pediatric patients assessed during attack-free periods, SAA has been reported to remain elevated more frequently than CRP and may therefore be a more sensitive marker of occult inflammation (4). Consequently, a single CRP measurement obtained during an attack-free outpatient visit may not comprehensively represent inter-attack inflammatory activity. Exertional leg pain was more frequent among patients with less favorable food-choice behavior. Exertional leg pain is a recognized musculoskeletal manifestation of FMF and has been associated with higher disease-severity scores, more frequent arthritis and arthralgia, increased colchicine requirements, and M694V variants in pediatric cohorts (32). Therefore, the association observed in our study may reflect differences in underlying disease phenotype, genetic background, physical activity, body composition, or other unmeasured factors rather than a direct effect of dietary behavior. BMI was also higher among patients with less favorable food-choice behavior, although the proportion of patients with a BMI percentile of ≥85% did not differ significantly between the FBS groups. This finding is consistent with the construct assessed by the FBS, which evaluates choices between foods differing primarily in fat and sodium content (22, 23). Nevertheless, the cross-sectional design prevents determination of the temporal direction of this relationship. Total energy intake, physical activity, pubertal status, socioeconomic characteristics, and parental dietary influence were not measured and may have affected both BMI and food-choice behavior. The associations involving BMI and exertional leg pain should therefore be regarded as exploratory.

A strength of our study was the concurrent use of two complementary dietary instruments. KIDMED reflects adherence to an overall Mediterranean dietary pattern, whereas the FBS evaluates behavioral choices between foods differing primarily in fat and sodium content (20–23). Their combined use enabled different dimensions of dietary behavior to be evaluated within the same pediatric FMF cohort. Conducting both questionnaire and laboratory assessments during an attack-free period also reduced the immediate influence of acute inflammatory episodes on dietary behavior, clinical symptoms, and inflammatory parameters. In addition, the detailed evaluation of *MEFV* genotype combinations and the presentation of their distribution improved the transparency of the genetic classification. These unmeasured variables may have influenced dietary scores, BMI, inflammatory parameters, and symptom reporting. Nevertheless, this study also has several limitations. First, its cross-sectional and single-center design limits causal interpretation and generalizability and may have introduced selection bias by reflecting the characteristics of a specific referral population. Second, dietary assessment was questionnaire-based and may have been affected by recall error, parental influence, and social desirability bias. The absence of a healthy control group also prevented determination of whether the observed patterns differed from those of otherwise healthy children or were specific to pediatric FMF. In addition, individual nutrient intake, total energy consumption, physical activity, socioeconomic status, and pubertal stage were not quantitatively assessed. These unmeasured variables may have influenced dietary scores, BMI, inflammatory parameters, and symptom reporting. Inflammatory evaluation was restricted to leukocyte count and CRP and did not include SAA, IL-1β, and IL-6, or repeated measurements during follow-up. The sample size may also have been insufficient to detect small differences, particularly in genotype and other subgroup analyses. Because of the limited sample size, multivariable adjustment for genotype and other potential confounders was not performed. Multiple secondary and exploratory comparisons were performed without adjustment across the complete family of analyses, increasing the risk of type I error. Accordingly, the findings involving leukocyte count, BMI, exertional leg pain, and genotype distribution should be regarded as exploratory. Concomitant anti-IL-1 therapy in patients with colchicine-resistant disease may also have influenced inflammatory biomarkers and clinical disease expression. The present analysis focused on the validated composite KIDMED and FBS scores and did not examine individual questionnaire items. A dedicated, adequately powered analysis would be required to evaluate whether specific food choices are associated with disease manifestations. Furthermore, although abdominal pain was evaluated as an FMF-related manifestation, other gastrointestinal symptoms, including diarrhea, constipation, nausea, vomiting, and abdominal bloating, were not systematically recorded. This limited the ability to assess the relationship between dietary patterns and gastrointestinal symptom burden comprehensively.

In pediatric patients with FMF, Mediterranean diet adherence and food-choice behavior were not associated with attack frequency or overall disease severity. Nominal exploratory associations were observed with BMI, exertional leg pain, and leukocyte count. In addition, M694V-containing genotypes were distributed unequally across the KIDMED categories, emphasizing the need to consider genetic background when interpreting diet-related findings. These findings do not establish a causal effect of dietary behavior on clinical or inflammatory disease expression. Larger prospective, multicenter studies incorporating detailed dietary assessment, repeated inflammatory measurements, physical activity and socioeconomic variables, and genotype-adjusted analyses are required to clarify the potential clinical relevance of dietary patterns in pediatric FMF.

## Data Availability

Due to institutional privacy policies and ethical considerations, the datasets are not publicly available but can be provided upon reasonable request from the corresponding author. Requests to access these datasets should be directed to ilgazhande92@gmail.com.
