# Supplementary material for: Mediterranean diet adherence in pediatric familial Mediterranean fever: clinical and inflammatory correlates
Source: Front Pediatr. 2026 Aug 11;14:1917680. doi: 10.3389/fped.2026.1917680 (PMC13503621; doi:10.3389/fped.2026.1917680)
Supplement: Supplementary file 1 [file Table1.docx]

**Supplementary Table S1.** Detailed distribution of *MEFV* genotype combinations included in the four-level genotype classification

| **Genotype category** | **Specific genotype combination** | **n (%)** |
| --- | --- | --- |
| Single-variant genotype | R202Q | 8 (8.8) |
|  | M694V | 7 (7.7) |
|  | E148Q | 5 (5.5) |
|  | M680I | 4 (4.4) |
|  | R761H | 2 (2.2) |
|  | A744S | 1 (1.1) |
|  | M694I | 1 (1.1) |
|  | P369S | 1 (1.1) |
| Homozygous genotype | M694V/M694V | 12 (13.2) |
|  | R202Q/R202Q | 4 (4.4) |
|  | V726A/V726A | 1 (1.1) |
| Two-variant genotype | M694V + R202Q | 8 (8.8) |
|  | M680I + M694V | 4 (4.4) |
|  | M694V + V726A | 3 (3.3) |
|  | M694V + R761H | 3 (3.3) |
|  | E148Q + R202Q | 2 (2.2) |
|  | E148Q + M680I | 1 (1.1) |
|  | E148Q + M694V | 1 (1.1) |
|  | E148Q + P369S | 1 (1.1) |
|  | E148Q + V726A | 1 (1.1) |
|  | M680I + R761H | 1 (1.1) |
|  | M680I + V726A | 1 (1.1) |
|  | M694V + T681I | 1 (1.1) |
|  | R202Q + V726A | 1 (1.1) |
| Complex/multivariant genotype | M694V/M694V + R202Q/R202Q | 7 (7.7) |
|  | M694V/M694V + R202Q | 6 (6.6) |
|  | A744S + M694V + R202Q | 1 (1.1) |
|  | E148Q + M694V + R202Q | 1 (1.1) |
|  | M694V + R202Q + V726A | 1 (1.1) |
|  | M490T + R204H + R761H | 1 (1.1) |

Data are presented as n (%), with percentages calculated using the overall cohort of 91 patients. Genotype combinations were grouped as single-variant, homozygous, two-variant, or complex/multivariant genotypes. The single-variant category indicates that only one *MEFV* variant was documented and should not necessarily be interpreted as confirmed heterozygosity. For combinations containing two different variants, the plus sign indicates coexistence of the variants and does not establish allelic phase or confirmed compound heterozygosity. The complex/multivariant category included genotypes containing three or more reported variant alleles or multiple zygosity combinations, including double homozygosity.
